# Emergency management: penetrating eye injuries and intraocular foreign bodies

**Published:** 2018-11-09

**Authors:** Nyawira Mwangi, Dorothy M Mutie

**Affiliations:** 1 Research Fellow: London School of Hygiene and Tropical Medicine, London, UK.; 2 Ophthalmologist/Lecturer: Kenya Medical Training College, Nairobi, Kenya.

**Penetrating injuries require immediate first aid and urgent referral to a specialist, particularly if there is a foreign body in the eye.**

**Figure:**
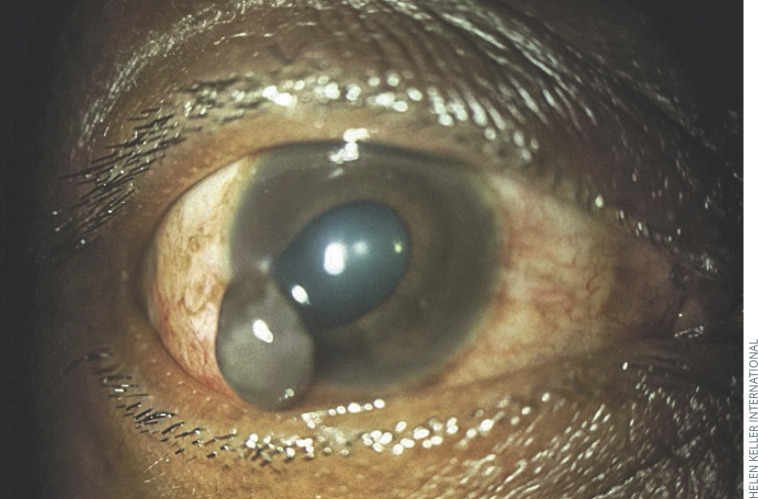
A penetrating eye injury with iris prolapse.

Up to 40% of penetrating eye injuries are complicated by the presence of an intraocular foreign body (IOFB).^1^,^2^ It may be toxic (iron, copper, vegetable matter) or inert (glass or plastic). Vision loss may result from the mechanical injury or from post-traumatic complications such as endophthalmitis, retinal detachment, metal toxicity and sympathetic ophthalmia.^2^ Prompt diagnosis, referral, removal of the IOFB and surgical repair will help to preserve the visual acuity and the globe anatomy.

## Recognition and diagnosis

When the patient presents, conduct an initial trauma assessment and resuscitation (if necessary) using the airway-breathing-circulation-disability-exposure (ABCDE) systematic approach.^3^ Ask about systemic comorbidities, allergies to medication and time of last meal. Non-ocular injuries, such as head injuries, should be managed with the help of other specialists.

What to ask when taking a history (ATMIST-V):

**Table:** 

**A**	**A**ge of patient
**T**	**T**ime and date of injury
**M**	**M**echanism (sharp object, hammer and chisel, sawing, grinding, explosives, broken windscreen, etc) and setting (work, home, gardening, assault, motor accident etc)
**I**	**I**njuries sustained
**S**	**S**ymptoms and signs (pain, redness, decreased vision)
**T**	**T**reatment or intervention already given
**-**	
**V**	**V**isual status before injury (previous history and surgery) and whether any protective eye wear was worn

A retained IOFB in the posterior segment is usually hidden from view (occult). Assume that the patient has a retained IOFB until proven otherwise, even after a long period of time.^1^,^2^

Record the baseline best-corrected visual acuity in each eye and conduct a complete examination of both eyes and adnexae. Use Desmarre's retractor to avoid undue pressure on the globe during examination.

History and clinical findings that should raise the suspicion of an IOFB include: a history of hammering a metal object, a scleral wound with uveal prolapse, a corneal entry point with oedema, a shallow anterior chamber, an iris hole, an irregular pupil, a lens defect and vitreous haemorrhage.

## Remedies and immediate management

After examination, you should:

1. Protect the eye from further damage by using an eye shield.
2. Administer systemic analgesics.
3. Administer prophylactic broad-spectrum systemic antibiotics.
4. Administer anti-emetics if the patient has nausea or vomiting.
5. Update tetanus prophylaxis.
6. Recommend ‘nil by mouth’ status in preparation for surgery.
7. Carefully document all findings and actions taken.

### Take note:

- Defer IOP measurements in patients with lacerations
- Avoid any pressure on the globe; for example, do not press on the sclera
- **Do not** attempt to pull out any foreign material that may be sticking out of the eye.

## Referral

Refer the patient **urgently** to a facility that has the following:

- An ophthalmic surgeon who is equipped for pars plana vitrectomy (required for posterior IOFB)
- Imaging facilities: orbital X-ray and ultrasound, and CT scan. MRI is contraindicated until you have excluded the possibility of a metallic IOFB
- An operating theatre where urgent removal of IOFB, intravitreal antibiotic injection and surgical repair can be done.

Gently explain to the patient that multiple operations may be required and that visual prognosis is uncertain, but taking up the referral as quickly as possible will give them the best chance. Send the patient with a comprehensive referral note and alert the surgeon.

## Rehearsal

In preparation for handling such a patient, you need to ensure that your clinic has the following items:

- **Equipment.** Desmarre's eye retractor, rigid eye shield, tape, standard examination equipment
- **Drugs.** Analgesics, antibiotics, anti-emetics, tetanus vaccine
- **Information.** Contact details of the nearest referral centre that can provide vitrectomy surgery.

Practise the following:

- Taking consent (and assent from children)
- Speaking with the patient about the visual prognosis
- Writing a referral note.
